# Complete Genome Sequence of the Fruiting Myxobacterium *Myxococcus macrosporus* Strain DSM 14697, Generated by PacBio Sequencing

**DOI:** 10.1128/genomeA.01127-17

**Published:** 2017-10-05

**Authors:** Anke Treuner-Lange, Marc Bruckskotten, Oliver Rupp, Alexander Goesmann, Lotte Søgaard-Andersen

**Affiliations:** a Department of Ecophysiology, Max Planck Institute for Terrestrial Microbiology, Marburg, Germany; b Bioinformatics and Systems Biology, Justus-Liebig University Giessen, Giessen, Germany

## Abstract

Members of the *Myxococcales* order initiate a developmental program in response to starvation that culminates in formation of spore-filled fruiting bodies. To investigate the genetic basis for fruiting body formation, we present the complete 8.9-Mb genome sequence of *Myxococcus macrosporus* strain DSM 14697, generated using the PacBio sequencing platform.

## GENOME ANNOUNCEMENT

Most members of the *Myxococcales* order initiate a developmental program in response to starvation that results in the formation of a multicellular fruiting body inside which cells differentiate to spores (1, 2). Analyses using *Myxococcus xanthus* as a model organism have provided important insights into regulation of fruiting body formation (3, 4). However, comparative genome investigations of different *Myxococcales* genome sequences have indicated that the developmental program that results in fruiting body formation is not highly conserved (5–7).

Only 20 genomes of the *Myxococcales* have been completely sequenced (5, 8–24). In addition, 36 *Myxococcales* draft genomes are available (25–32). To generate additional resources for accurate genomic comparisons and eventually decipher and compare the genetic programs for fruiting body formation, we sequenced and annotated the complete genome of *Myxococcus macrosporus* strain DSM 14697, which was obtained from the Deutsche Sammlung von Mikroorganismen und Zellkulturen GmbH.

After verification of the formation of haystack-shaped fruiting bodies by *M. macrosporus* DSM 14697, we collected genomic DNA (33) and sequenced it using PacBio single-molecule real-time (SMRT) sequencing (34) on the PacBio RSII platform at the Max Planck-Genome-Centre Cologne, Germany. Two SMRT cells were used. After quality evaluation and filtering of 184,213 subreads, the assembly process using the HGAP assembly pipeline (35) resulted in one contig with 83-fold coverage, which allowed a manual closure of the contig. The genome was verified for completion and oriented with DnaA as the first locus tag. Genome annotation was done using Prokka (36). BLASTP searches against the RefSeq database were used to assign functional annotation and identify possible frameshifts in genes. The corresponding genes were removed from the annotation.

The complete genome sequence of *M. macrosporus* DSM 14697 contains 8,973,512 bp with a GC content of 70.6%. A total of 7,143 protein-coding sequences (CDSs) were identified together with 79 tRNA genes and 12 rRNA operons. The size of the *M. macrosporus* genome is similar to those of other sequenced genomes of fruiting myxobacteria, which range in size from 9.0 Mb to 16.0 Mb. Aligning the *M. macrosporus* genome with other completely sequenced *Myxococcales* genomes by using NUCmer (37) revealed overall synteny, particularly to other *Myxococcus* species in the following order (% alignment): *Myxococcus fulvus* HW-1 (93.5), *M. xanthus* DK 1622 (80.6), *M. hansupus* (73.4), *M. fulvus* 124B02 (43.9),* and M. stipitatus* DSM_14675 (38.3). The best matches outside the genus *Myxococcus* are to *Corallococcus coralloides* DSM_2259 (31.4) and *Archangium gephyra* DSM_2261 (20.3).

The *M. macrosporus* genome sequence will contribute to the investigation of the genetic programs leading to fruiting body formation.

### Accession number(s).

The genome sequence was deposited in GenBank under accession number CP022203.
